# Bi-Hemispheric Adversarial Domain Adaptation Neural Network for EEG-Based Emotion Recognition

**DOI:** 10.3390/brainsci16050507

**Published:** 2026-05-08

**Authors:** Yuqi Chen, Ming Meng

**Affiliations:** 1 School of Hangzhou Dianzi University ITMO Joint Institute, Hangzhou Dianzi University, Hangzhou 310027, China; 232320074@hdu.edu.cn; 2 School of Automation, Hangzhou Dianzi University, Hangzhou 310018, China

**Keywords:** emotion recognition, EEG signal, adversarial domain adaptation, class-informed discriminator

## Abstract

**Highlights:**

**What are the main findings?**

• This study proposes a **class-informed discriminator**. Instead of using the conventional binary domain discriminator in DANN that only distinguishes source and target domains, the proposed method incorporates source domain class structure into the discrimination process and extends the discriminator to a (C + 1) classification setting. This design helps enforce intra-class alignment across domains while reducing inter-class confusion, thereby alleviating the category mismatch problem commonly encountered in adversarial domain adaptation.
• This study proposes the **BiHADA framework**, which independently models the left and right hemispheres and combines their predictions through a perplexity-based weighted decision strategy. By separately constructing feature extractors, discriminators, and classifiers for each hemisphere, the method captures hemispheric asymmetry in emotional processing more effectively. In addition, perplexity is introduced to evaluate the domain alignment quality of each hemisphere branch and to adaptively assign their decision weights, improving the robustness and accuracy of the final classification.

**What are the implications of the main findings?**

• The proposed class-informed discriminator shows that domain alignment in EEG emotion recognition should not only reduce the overall distribution discrepancy between domains, but also preserve category-level discriminative structure. This provides a useful direction for designing more reliable adversarial adaptation methods under cross-subject and cross-session settings.
• The proposed BiHADA framework indicates that incorporating hemispheric functional differences and branch-wise adaptation quality into the decision process can further improve cross-domain EEG emotion recognition. This offers a new perspective for building more interpretable and robust models by combining neurophysiological priors with adaptive weighting strategies.

**Abstract:**

**Background/Objectives:** Adversarial domain adaptation methods are widely used in EEG-based emotion recognition to reduce the influence of individual differences and the non-stationary characteristics of electroencephalogram (EEG) signals. Most existing methods employ binary domain discriminators to align source and target domains at the global distribution level. However, such strategies often neglect the potential multimodal structure of emotional EEG data and the asymmetric emotional processing characteristics of the left and right hemispheres. To address these issues, this study proposes a Bi-Hemispheric Adversarial Domain Adaptation Neural Network (BiHADA) for EEG-based emotion recognition. **Methods:** In the proposed BiHADA framework, the conventional binary domain discriminator is extended into a multimodal discriminator by incorporating the label structure information of source-domain data into the domain discrimination process. This design encourages features belonging to the same emotional category to be aligned across domains and promotes positive knowledge transfer. In addition, dual adversarial domain adaptation branches are constructed to model the left and right hemispheres separately, enabling the network to capture hemisphere-specific emotional representations. Furthermore, discriminator-derived perplexity is introduced to evaluate the distribution alignment quality of target samples and to adaptively determine the weights of the corresponding hemisphere classifiers, thereby reducing the influence of poorly aligned samples during the final decision stage. **Results:** Experiments on the SEED dataset show that BiHADA achieves classification accuracies of 86.82% and 92.71% in cross-subject and cross-session tasks, respectively. These results demonstrate that the proposed method can effectively improve the transferability and discriminability of EEG emotional features under different domain adaptation scenarios. **Conclusions:** The proposed BiHADA method enhances EEG-based emotion recognition by jointly considering class-structure-guided domain alignment, hemispheric functional asymmetry, and branch-wise adaptation quality. The results suggest that incorporating source-domain label structure and hemisphere-specific adaptation can improve cross-domain EEG emotion recognition performance.

## 1. Introduction

Emotions are fundamental to human communication and behavior, and accurately recognizing them is crucial for understanding human actions and enhancing human–computer interaction. With the evolution of deep learning methods combined with breakthroughs in neuroscience, researchers have begun to focus on implementing electroencephalogram (EEG) signals for emotion recognition. EEG signals have high temporal resolution, making them capable of providing rich information of emotions. Researchers can more accurately decode emotional information, thus bringing breakthroughs to the field of emotion recognition. This progress offers more effective solutions for social interaction and mental health. Consequently, emotion recognition based on EEG signals has become a highly sought-after research topic.

Initially, researchers conducted studies on a single subject or a single session. Xiang et al. [1] introduced Convolutional Recurrent Neural Networks (C-RNNs) to extract task-specific features and incorporate contextual information from the frames. Liu et al. [2] proposed a Bimodal Deep AutoEncoder (BDAE) to extract high-level features from EEG signals as well as other physiological signals. Li et al. [3] introduced Hierarchical Convolutional Neural Networks (HCNNs) to construct a 2D representation of EEG signals based on the spatial positions of the electrodes. These representations serve as inputs to the hierarchical convolutional network. Despite achieving high emotion classification accuracy, the aforementioned methods are typically trained on data from a single subject and within a single session. However, emotion recognition models require robust generalization capabilities in cross-domain scenarios.

Domain adaptation (DA) aims to transfer the knowledge learned from the source domain to improve the performance of the model in the target domain, which is a promising solution in tackling the concerns of high heterogeneity of EEG signals. Zhang et al. [4] used maximum mean discrepancy (MMD) to quantify the similarities between the source subjects and the target subject. Chai et al. [5] proposed adaptive subspace feature matching to reduce the distribution discrepancy. Domain adaptation technology is currently widely used in emotion recognition tasks across subjects and sessions. It can reduce the differences in EEG feature distributions between different subjects and sessions. EEG features with cross-domain invariance can enhance the generalization ability of emotion recognition models and improve their classification performance in the target domain [6]. However, traditional domain adaptation methods rely on explicit metrics to align the feature distributions between the source domain and the target domain. These methods usually only focus on the alignment of feature distributions and may not be able to effectively capture more complex structural features or nonlinear relationships.

**Figure 1:**
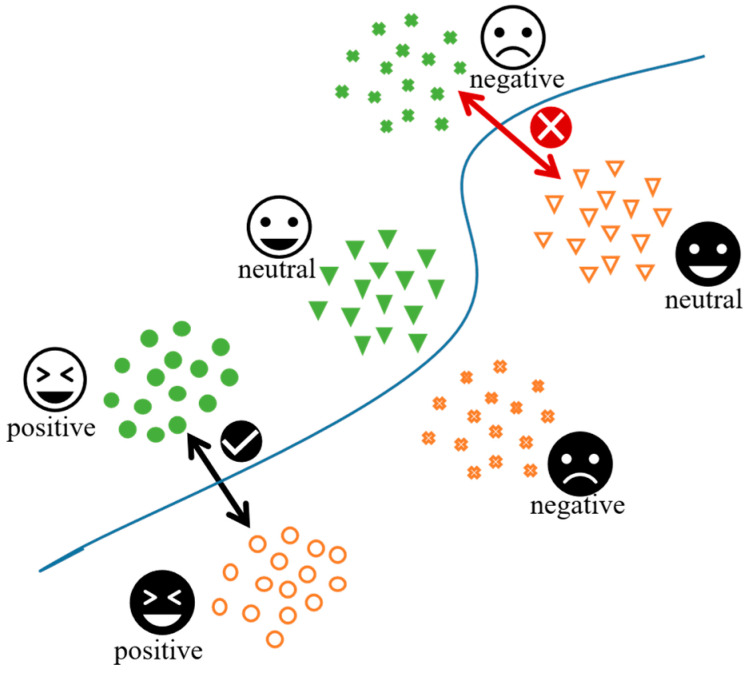
Misalignment of cross-domain discriminative structures. Green samples denote source-domain data, while orange samples denote target-domain data. Different marker shapes represent different emotion categories: circles indicate positive emotion, triangles indicate neutral emotion, and crosses indicate negative emotion. The arrows illustrate the alignment tendency between target-domain samples and source-domain emotion categories, where correct class-aware alignment helps reduce cross-domain discrepancy, whereas incorrect alignment may lead to category confusion.

Generative Adversarial Networks (GANs) adopt an adversarial learning strategy, in which feature extractors and domain discriminators confront each other [7]. The key advantage of this method is that it does not require the manual design of complex alignment metrics—the model can learn appropriate feature representations through an unsupervised training manner. Inspired by GANs, Ganin et al. [8] introduced the Domain-Adversarial Neural Network (DANN), which incorporates an additional domain discriminator to determine whether the extracted features originate from the source or target domain. Utilizing a gradient reversal layer, the extractor is encouraged to produce features which are indistinguishable at the domain level. Some researchers have used DANN to align data distributions across various subjects or sessions in the context of EEG emotion recognition [9]. Although DANN has shown good classification performance in cross-subject emotion EEG recognition, DANN’s adversarial domain adaptation process can sometimes degrade the classifier’s performance because it focuses solely on the overall alignment of domain distributions neglecting intricate multimodal structures. During adversarial training, not only are the samples from both domains confused, but the category discriminative structures of the data may also be misaligned, which results in the incorrect alignment of discriminative structures from different distributions, as illustrated in Figure 1. 

From a neuroscience perspective, the human brain exhibits partial asymmetry between left and right hemispheres, and its response to emotions is similarly asymmetrical. Dimond et al. [10] were the first to discover that different hemispheres of the human brain possess unique “emotional visions,” with the right hemisphere having a stronger perception of unpleasant emotions. Davidson et al. [11,12] further demonstrated that EEG signals from the left frontal cortex are closely associated with positive emotions, while those from the right frontal cortex are more closely related to negative emotions. This has prompted researchers to leverage the asymmetry between both hemispheres in emotion recognition studies. Lingelbach et al. [13] used fNIRS in a naturalistic setting and found that emotional language interference and cognitive load interact to produce different left–right PFC activation patterns, with the effect varying by load level. Chmiel et al. [14] reported that individuals with alexithymia exhibit stronger right-hemisphere activity across multiple frequency bands, including alpha and theta waves, when exposed to emotional stimuli, along with reduced connectivity, indicating a more prominent role of the right hemisphere in emotional processing.

Zhong et al. [15] introduced a Transformer-based Bi-Hemispheric Asymmetric Attention Network (Bi-AAN). This model combines the Transformer structure and brain’s asymmetric emotional response characteristics. It employs a dual-headed attention mechanism to capture internal attention within each hemisphere’s frequency band and attention differences between frequency bands across both hemispheres. Huang et al. [16] introduced a Bi-hemisphere Discrepancy Convolutional Neural Network (BiDCNN), designed to capture differing response patterns between both hemispheres. The model has three inputs and one output, utilizing three convolutional neural network layers. Three distinct feature matrices are created and processed through the layers to extract spatial and temporal features. Li et al. [17] proposed a Bi-Hemisphere Domain Adversarial Neural Network (BiDANN) model, which consists of a classifier and three domain discriminators, with adversarial tasks between them. Two local domain discriminators correspond to each hemisphere of the brain, facilitating the learning of discriminative emotional features locally within each hemisphere. Another global domain discriminator attempts to minimize the overall differences between the domains. Although BiDANN introduces hemisphere-specific domain discriminators, its discriminators still perform binary source–target discrimination. Therefore, the adaptation process mainly reduces the global or local domain discrepancy without explicitly modeling the category structure of emotional EEG features.

To improve the discriminative capabilities on class-level of domain adaptation in bi-hemispheric model, this study introduces the concept of making the source domain label structure accessible to a binary discriminator, aiming to uncover the potential multimodal information within the data. This approach constructs a class-informed discriminator to avoid the loss of feature discriminative information and facilitate the correct alignment of cross-domain features from the same category. Additionally, this model constructs two independent adversarial domain adaptation network branches for both hemispheres separately. This exploration aims to investigate the perception capabilities of different brain hemispheres for specific emotions and to comprehensively utilize the domain adaptation levels of the left and right brain hemispheres to further advance emotion EEG recognition research.

## 2. Materials and Methods

**Figure 2:**
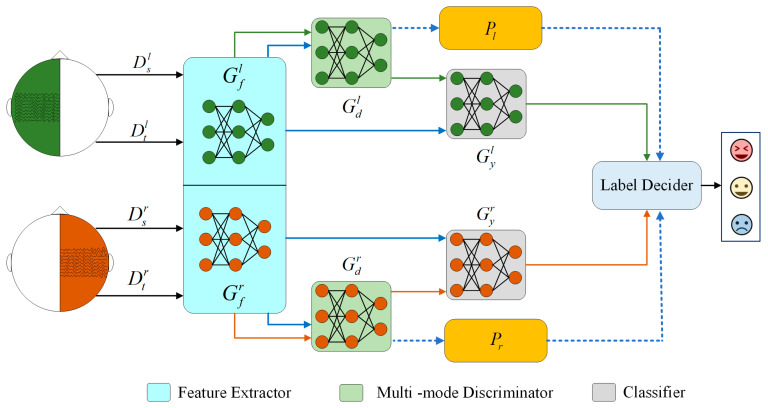
The architecture of the Adversarial Adaptation Neural Networks with Class-informed Discriminator.

BiHADA constructs independent feature extractors and discriminators for the left and right brain hemispheres, and incorporates source domain label information to build a class-informed discriminator, enabling class-level cross-domain alignment. Meanwhile, a perplexity-based mechanism is introduced to dynamically weight the classification outputs from both hemispheres, thereby improving the accuracy and robustness of emotion recognition. The structure of BiHADA is illustrated in Figure 2.

### 2.1. Class-Informed Discriminator

In the DANN framework, a binary domain label discriminator directs the extractor module to obtain domain-invariant feature representations at the domain level via reverse optimization. This framework ensures that the features have some transferability across domains. However, during training, the category label structure information is unknown to the binary discriminator. As a consequence, when target domain data are projected into the feature space, the absence of multimodal information results in the features being erroneously classified into one source domain, causing the discriminative information loss of target domain features during the domain adaptation process. In the process of aligning marginal probability distributions, this leads to the misalignment of samples from different categories across domains, thereby reducing the model’s classification performance.

From the perspective of distribution alignment, standard DANN mainly reduces the discrepancy between the marginal feature distributions of the source and target domains, i.e., $P_{s}(f)$ and $P_{t}(f)$. However, EEG emotion features usually exhibit class-dependent multimodal structures, where each emotion category corresponds to a specific feature sub-distribution. Therefore, aligning only the marginal distributions cannot guarantee that samples with the same emotion label are aligned across domains. In some cases, marginal alignment may even pull target domain samples toward source domain samples from different emotion categories, leading to cross-category misalignment and degraded target domain classification performance. This indicates that cross-domain EEG emotion recognition requires not only marginal distribution alignment but also approximate class-conditional distribution alignment, namely reducing the discrepancy between $P_{s}(f|y)$ and $P_{t}(f|y)$.

The label structure of source domain samples can effectively reveal multiple data modes. Therefore, in this section, the label information of the source domain data is provided to the binary domain discriminator. By utilizing the emotional multimodal information revealed by the source domain label structure, a class-informed discriminator is constructed to expand the discriminative dimension. Its prediction output $p\in R^{C+1}$ corresponds to C + 1 categories, including the emotional categories contained in the source domain label structure and an additional target domain category $d_{t}$. The domain label $d_{s}$ of the source domain is replaced with specific emotional category labels. During adversarial training, this class-informed discriminator performs class-level discrimination on the data from both domains. When extractor module deceives the discriminator, it further induces the discriminator to misclassify target domain samples into specific classes of the source domain. By learning from the multimodal information revealed by the source domain data, more accurate distribution alignment at the class level is achieved.

In the realm of emotion recognition, the label structures of samples from both domains are typically consistent. The label information from the source domain can provide highly effective multimodal information in such a closed set situation [18]. This helps guide target domain samples to transform into a more suitable feature space, thereby enhancing the model’s classification accuracy.

It should be emphasized that the proposed class-informed discriminator is not a simple modification of the discriminator output dimension. Its key role is to change the adaptation criterion from domain-level indistinguishability to category-structure-preserving domain alignment. By assigning source samples to their emotion categories and target samples to an additional target domain class, the discriminator learns both domain separability and source domain class structure. During adversarial optimization, the feature extractor is encouraged to map target domain samples toward source domain emotional modes rather than merely confusing the two domains globally.

### 2.2. Adversarial Adaptation Neural Networks with Class-Informed Discriminator

**Figure 3:**
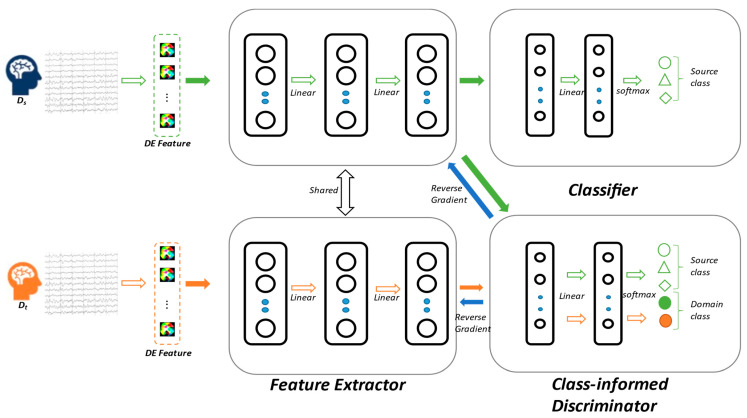
The architecture of the Adversarial Adaptation Neural Networks with Class-informed Discriminator. The network consists of a feature extractor, a task-specific independent classifier, and a class-informed discriminator.

We provide a detailed introduction to the overall framework of the proposed Adversarial Domain Adaptation Neural Network with Class-Informed Discriminator (ADA-CiD). The original EEG signals are processed into differential entropy (DE) feature vectors as the model input. The model consists of three main modules: the feature extractor $G_{f}(\cdot ;\theta_{f})$ with parameters $\theta_{f}$, the classifier $G_{y}(\cdot ;\theta_{y})$ with parameters $\theta_{y}$, and the class-informed discriminator $G_{d}(\cdot ;\theta_{d})$ with parameters $\theta_{d}$. The structure of BiHADA is illustrated in Figure 3.

The DE features, which are high-dimensional vectors, serve as the input to the model, and a Multi-Layer Perceptron is employed to extract EEG emotion features. During training, both source and target domain samples share a common feature extractor, $G_{f}$. After feature extraction by the Multi-Layer Perceptron (MLP), the source domain features are supplied to classifier $G_{y}$ and discriminator $G_{d}$. The classifier performs a classification over C categories, corresponding to the source domain labels, while the discriminator performs a classification of C + 1 categories, where the extra category indicates whether a sample comes from the source domain or target domain. The ADA-CiD model does not require the classifier to generate pseudo-labels for the target domain samples during training.

The gradient reversal layer adjusts the weights of the feature extractor during backpropagation, aiming to extract target domain features that can deceive the discriminator as much as possible. This encourages the discriminator to classify target domain samples into specific emotional categories rather than domain categories. The label structure information helps the model learn various underlying modes in the classification task. By adversarial relationships, class-level domain adaptation is achieved instead of mere domain-level alignment. This effectively prevents the misalignment of features from different categories in both domains during adaptation process, thus reducing the loss of feature discriminative information.

The classic discriminator loss is defined as follows:

(1) $$L_{d}\left(\theta_{f},\;\theta_{d}\right)=L_{\mathrm{ce}}\left(G_{d}\left(G_{f}\left(x_{i};\theta_{f}\right);\theta_{d}\right),\;d_{i}\right)$$

where $L_{ce}$ represents the cross-entropy loss, and $d_{i}$ is a binary label of domains defined as

(2) $$d_{i}=\left\{\begin{array}{ll} 1, & x_{i}\in D_{s}\\ 0, & x_{i}\in D_{t} \end{array}\right.$$

In the classical binary discriminator modules, binary classification is performed solely at the domain level, learning from visible domain labels. This approach achieves domain-level domain adaptation. However, during this process, the discriminator lacks visibility into the category structure, leading to feature transformations lacking guided mode information. Consequently, target domain features lack necessary discriminative structures, resulting in an inability to ensure feature specificity within the domain. As a consequence, classifiers may struggle to correctly differentiate between categories of target domain samples, leading to a decrease in model generalization capability. Contrarily, in the proposed ADA-CiD model in this section, the label structure information from the source domain is visible to the discriminator. Therefore, post-learning, the discriminator can utilize the multimodal information provided by the source domain data to predict specific categories of input features, rather than solely performing binary classification at the domain level. Adversarial training encourages the extracted features to be indistinguishable across domains while also preserving their specificity within the domain. Moreover, since emotion EEG tasks typically fall under closed-set DA tasks, the effectiveness of source domain class label information in guiding target domain feature transformations is partially guaranteed.

Consequently, the class-informed discriminator loss in ADA-CiD can be rewritten as follows:

(3) $$L_{\mathrm{dc}}\left(\theta_{f},\;\theta_{d}\right)=L_{\mathrm{ce}}(G_{d}\left(G_{f}\left(x_{i};\theta_{f}\right);\theta_{d}\right),\;{\mathrm{dc}}_{i})$$

where ${dc}_{i}$ represents the domain label, with $D_{s}$ using specific class labels for the source domain, and $D_{t}$ corresponding to an additional target domain label.

(4) $${\mathrm{dc}}_{i}=\left\{\begin{array}{c} y_{i}\;,\;\;\;\;\;\;\;\;\;\;\;x_{i}\in D_{s}\\ C+1,\;\;\;\;\;\;\;x_{i}\in D_{t} \end{array}\right.$$

where $y_{i}$ is the class label obtained from the class-informed discriminator, and C denotes the number of emotion classes.

The classifier module consists of two fully connected layers. During training, the classifier only needs to receive representations of source domain features outputted by the feature extractor. After making predictions, it combines these predictions with the true labels to compute the cross-entropy loss for further optimization. The classifier aims to achieve high accuracy in classifying the source domain data. It is important to note that while the discriminator has access to source domain label information and also performs classification tasks, its classification is aimed at guiding the domain adaptation process through the gradient reversal layer. Ultimately, its goal is to find a feature space that aligns the features from different domains. The loss function for the classifier is defined as follows:

(5) $$L_{y}(\theta_{f},\;\theta_{y})=L_{\mathrm{ce}}(G_{y}(G_{f}(x_{i};\theta_{f});\theta_{y}),\;y_{i})$$

During the training phase, this loss consists of two components. The first component is the loss of the classifier for source samples, and the other part is the discrimination loss of the discriminator for both domains.

(6) $$\;L\left(\theta_{f},\theta_{y},\theta_{d}\right)=\frac{1}{n_{s}}\sum_{i=1}^{n_{s}}L_{y}\;\left(G_{y}\;\left(G_{f}\left(x_{i}^{s}\right)\right),y_{i}^{s}\right)+\frac{\lambda }{n_{s}+n_{t}}\sum_{i=1}^{n_{s}+n_{t}}L_{\mathrm{dc}}\;\left(G_{d}\;\left(u_{f}\left(x_{i}\right)\right),d_{c_{i}}\right)$$

where λ represents the weighting parameter balancing the two parts of the loss, $L_{y}$ and $L_{\mathrm{dc}}$ represents the cross-entropy losses of classifier $G_{y}$ and multimodal discriminator $G_{d}$ respectively. 

### 2.3. Bi-Hemispheic Adversarial Domain Adaptation Neural Network

The human brain exhibits an incompletely symmetric characteristic between its left and right hemispheres, with each hemisphere possessing lateralization mechanisms for emotions, resulting in differential emotional responses to external stimuli. Due to varying emotional modes, the perceptual abilities of brain regions differ, and modeling both hemispheres indiscriminately can affect their perceptual abilities for specific emotions. Addressing the incomplete symmetry of the brain, this study constructs a bi-hemispheric adversarial domain adaptation network. Utilizing the model ADA-CiD proposed in the previous section, the left and right hemispheres of the brain are individually modeled, forming independent branches of adversarial domain adaptation networks. This approach investigates the perceptual abilities of both hemispheres for specific emotions, while simultaneously leveraging degree of domain adaptation of both hemispheres to further explore emotional EEG recognition. The model proposed in this section constructs separate branch networks for the left and right hemispheres. For the adversarial domain adaptation modules of each hemisphere, the objective remains to minimize the distribution differences between both domains.

Due to the different electrodes corresponding to different hemispheres, it is necessary to divide features according to electrodes and extract the differential entropy features corresponding to the left and right hemisphere electrodes separately. Taking the SEED dataset with 62 channels as an example, firstly, the reference electrodes located in the midline are removed from the feature sequence according to the 10–20 system [19,20]. The removed electrode names are FPZ, FZ, FCZ, CZ, CPZ, PZ, POZ, and OZ, respectively. Based on the midline, each hemisphere contains 27 electrodes, which are concatenated and stacked horizontally to form the feature.

Modeling both hemispheres separately contributes to preserving their respective emotional visualizations, in accordance with the emotional lateralization mechanism of the brain. Utilizing the ADA-CiD model proposed in the previous section, separate adversarial domain adaptation branch networks are established for different hemispheres. Addressing asymmetry of the brain, a Bi-Hemispheric Adversarial Domain Adaptation Neural Network (BiHADA) is constructed. The architecture is illustrated in Figure 2. $D_{d}^{l}$ and $D_{d}^{r}$ represent the source and target domains for both hemispheres, respectively. $X_{s}^{l}$ and $X_{s}^{r}$ represent samples with shared source domain labels $Y_{s}$ in the left hemisphere source domain $D_{s}^{l}$ and the right hemisphere source domain $D_{s}^{r}$. $X_{t}^{l}$ and $X_{t}^{r}$ represent unlabeled samples in the left hemisphere target domain $D_{t}^{l}$ and the right hemisphere target domain $D_{t}^{r}$, defined as follows:

(7) $$X_{s}^{h}={\left\{x_{s}^{i}\right\}}_{i=1}^{m},\;X_{t}^{h}={\left\{x_{t}^{i}\right\}}_{i=1}^{n},\;Y_{s}={\left\{y_{s}^{i}\right\}}_{i=1}^{m}$$

where h denotes the hemisphere to which the data belongs, and m and n represent the data counts of both domains, respectively, within that hemisphere.

Using the feature extractor $G_{f}^{h}$ specific to each hemisphere, the source domain data $X_{s}^{h}$ and target domain data $X_{t}^{h}$ from the corresponding hemisphere are transformed into the feature space, with the feature representation defined as follows:

(8) $${X_{s}^{h}}^{\prime }=G_{f}^{h}\left(X_{s}^{h}\right),\;{X_{t}^{h}}^{\prime }=G_{f}^{h}\left(X_{t}^{h}\right)$$

The feature extractor $G_{f}^{h}$ is not shared between hemispheres because different hemispheres may have different emotional visualizations, meaning they may perceive emotions differently. Therefore, independent feature extractors are advantageous for extracting more separable features from data in different hemispheres. The deep feature representations obtained through $G_{f}^{h}$ are fed separately into the hemisphere-specific class-informed discriminator $G_{d}^{h}$ and classifier $G_{y}^{h}$ for mode discrimination and label prediction, respectively. The classifier module $G_{y}^{h}$ receives the feature representations from the corresponding hemisphere and performs emotion classification. Each hemisphere’s classifier $G_{y}^{h}$ is trained using data from the respective hemisphere’s source domain. The classifier $G_{y}^{h}$ outputs predicted labels ${Y_{s}}^{\prime }$ based on the feature representation ${X_{s}^{h}}^{\prime }$, as shown in the following equation:

(9) $${Y_{s}}^{\prime }=G_{y}^{h}\left({X_{s}^{h}}^{\prime }\right)$$

The class-informed discriminators corresponding to the left and right hemispheres respectively receive features from their respective hemispheres and perform discrimination. Through gradient reversal layers during backpropagation, they establish an adversarial relationship with extractors to align the marginal distributions for each hemisphere. The discriminators for specific hemispheres output predicted labels ${Y_{d,s}^{h}}^{\prime }$ and ${Y_{d,t}^{h}}^{\prime }$ based on the deep feature representations ${X_{s}^{h}}^{\prime }$ and ${X_{t}^{h}}^{\prime }$, as shown in the following equation:

(10) $${Y_{d,s}^{h}}^{\prime }=G_{d}^{h}\left({X_{s}^{h}}^{\prime }\right),\;{Y_{d,t}^{h}}^{\prime }=G_{d}^{h}\left({X_{t}^{h}}^{\prime }\right)$$

Unlike traditional domain discriminators, in the training process of BiHADA, source domain data along with their class labels are exposed to the discriminators, constructing class-informed discriminators. Therefore, the discriminators perform C + 1 classification, where C represents the count of source domain labels, and an additional class label represents the target domain.

The class-informed discriminators for the left and right hemispheres not only provide feedback to the domain-invariant feature extractor in their respective hemispheres via gradient reversal layers but also play the role of providing the perplexity of the target domain data to the classifier.

Since class-informed discriminators in the model have adequately learned the mode information of the EEG emotions through the label space structure of the source domain data in their respective hemispheres, the domain-invariant feature extractor will induce these discriminators to misclassify target domain features into specific emotional categories. Therefore, when the discriminator of a hemisphere accurately identifies target domain features, it indicates that the target domain features have failed to align with the features of the same emotional category in the feature space. Consequently, the consistency between different features exhibits substantial discrepancies, resulting in significant differences in the marginal probability distributions. Thus, classifiers trained on the corresponding hemisphere’s source domain data may struggle to effectively classify target domain features. Conversely, if the marginal probability distribution differences in that hemisphere are small, the classifier can perform well on the target domain. The perplexity of target domain samples, calculated using the C + 1 dimensional vector z outputted by the hemisphere’s discriminator, can be used to assess the degree of proximity between both domains of that hemisphere. The perplexity is defined as follows:

(11) $$P_{h}\left(z_{t}\right)\;=\;-\log\left(\frac{\exp\left(z_{t,d_{t}}\right)}{\sum_{i=1}^{C+1}\exp\left(z_{t,i}\right)}\right)$$

(12) $$d_{t}=C+1$$

where $z_{t}$ represents the output feature vector of the discriminator for target domain samples, $d_{t}$ represents the target domain labels, and C denotes the count of classes.

When the class-informed discriminator of a specific hemisphere cannot accurately predict the domain labels of target domain data, it generates a higher perplexity. Therefore, perplexity can be utilized to measure the domain adaptation degree of that hemisphere. Perplexity is then used to adjust the weights for the classifier of that hemisphere accordingly. The weight for the classifier corresponding to the discriminator of a specific hemisphere is defined as follows:

(13) $$W_{h}=\frac{P_{h}\left(z_{t}\right)}{P_{l}\left(z_{t}\right)+P_{r}\left(z_{t}\right)}$$

where $P_{l}$ and $P_{r}$ represent the perplexity of both hemispheres.

The label decider is used to determine the labels of target domain samples. It receives perplexity $P_{h}$ computed by the discriminator corresponding to the hemisphere, as well as the output of the classifier $G_{y}^{h}\left(G_{f}^{h}\left(x_{t}\right)\right)$ associated with that hemisphere. Based on the perplexity, the label decider assigns weights to the classifier of the corresponding hemisphere, and then combines the weighted classifiers to determine the comprehensive prediction result for the target domain data, as shown in the following equation:

(14) $$G_{y}\left(c\left|x_{t}\right.\right)=W_{l}G_{y}^{l}\left(c\left|G_{f}^{l}\left(x_{t}\right)\right.\right)+W_{r}G_{y}^{r}\left(c\left|G_{f}^{r}(x_{t})\right.\right)$$

where $W_{l}$ and $W_{r}$ represent the weights of the classifiers for both hemispheres, and $G_{y}^{l}$ and $G_{y}^{r}$ represent prediction results of the classifiers for the left and right hemispheres respectively. It should be noted that the perplexity is not a direct measure of general prediction uncertainty, but a proxy for alignment quality built upon the class-informed discriminator. Unlike a conventional binary domain discriminator, the class-informed discriminator models not only the source–target discrepancy but also the class structure of the source domain. Under this setting, if the adaptation quality of a hemisphere-specific branch is poor, the target domain features remain highly distinguishable from the source domain features, and the discriminator can still confidently assign them to the additional target domain category. In this case, the discriminator output tends to be more concentrated, leading to lower entropy and lower perplexity. In contrast, if a hemisphere-specific branch achieves better adaptation, the target domain features become closer to one or more source domain emotional classes in the learned feature space. As a result, the discriminator becomes less able to confidently classify them as the target domain category, and instead produces more competitive predictions among the target domain category and source domain emotion categories. This yields a more dispersed output distribution, corresponding to higher entropy and higher perplexity. Therefore, within the proposed class-informed discrimination framework, a higher perplexity is not merely a sign of model instability, but more importantly reflects the reduced domain separability and the increased proximity of target domain features to the source domain class structure. Based on this interpretation, perplexity is used as a proxy for branch-wise adaptation quality, and the corresponding classifier weights are dynamically assigned according to it.

The BiHADA model trains branch networks using labeled source domain data and unlabeled target domain data separately for the left and right hemispheres. The feature extractor and classifier for a specific hemisphere are trained using labeled source domain data. By constructing the adversarial relationship between the class-informed discriminator and the feature extractor within each hemisphere, the marginal probability distributions of the data in each hemisphere are aligned.

For a specific hemisphere, the classification loss is defined as follows:

(15) $${\;L}_{\mathrm{cls}}^{h}=\frac{1}{n_{s}}\sum_{x_{i}\in D_{s}^{h}}L_{\mathrm{ce}}(G_{y}^{h}(G_{f}^{h}(x_{i})),\;y_{i})$$

where $n_{s}$ denotes the count of source samples, $L_{ce}$ denotes cross-entropy loss, $G_{y}^{h}$ and $G_{f}^{h}$ respectively represent the classifier and feature extractor for the specific hemisphere, and $y_{i}$ represents the emotional label.

To minimize the misalignment of features from different categories, an ADA-CiD module is used. For each specific hemisphere, the discriminator loss $L_{\mathrm{adv}}^{h}$ is defined as follows:

(16) $$L_{\mathrm{adv}}^{h}=\frac{\lambda }{n_{s}+n_{t}}\sum_{i=1}^{n_{s}+n_{t}}L_{\mathrm{ce}}\left(G_{d}^{h}\left(G_{f}^{h}\left(x_{i}\right)\right),{\mathrm{dc}}_{i}\right)$$

where $n_{t}$ represents the target sample numbers, $G_{d}^{h}$ denotes the discriminator of the specific hemisphere, and λ serves as its weight parameter.

The overall loss function for each hemisphere’s branch network is defined as follows:

(17) $$\begin{array}{ll} L\left(\theta_{f},\;\theta_{y},\theta_{d}\right) & =\frac{1}{n_{s}}\sum_{i=1}^{n_{s}}L_{\mathrm{ce}}\left(G_{y}^{h}\left(G_{f}^{h}\left(x_{i}^{s}\right)\right),y_{i}\right)\\ & +\frac{\lambda }{n_{s}+n_{t}}\sum_{i=1}^{n_{s}+n_{t}}L_{\mathrm{ce}}\left(G_{d}^{h}\left(G_{f}^{h}\left(x_{i}\right)\right),{\mathrm{dc}}_{i}\right) \end{array}$$

Rather than merely splitting EEG channels according to hemispheric locations, the proposed bi-hemispheric design establishes an independent adversarial adaptation branch for each hemisphere. Each branch contains a dedicated feature extractor, class-informed discriminator, and emotion classifier, allowing hemisphere-specific transferable representations to be learned under separate adaptation processes. Moreover, this structure makes it possible to estimate the adaptation reliability of each hemisphere independently, thereby providing a basis for reliability-aware decision fusion.

## 3. Experiment

### 3.1. Dataset

**Figure 4:**
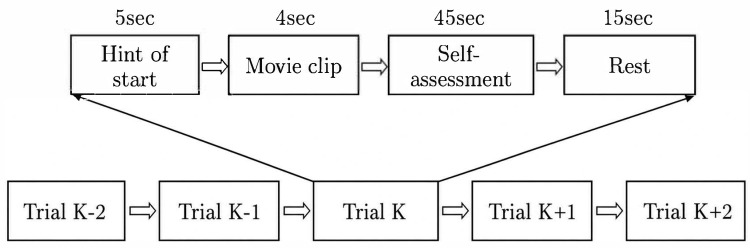
SEED data collection experimental paradigm.

This study uses the SEED [19,20], SEED-IV [21] and SEED-V [22] datasets to assess the proposed model, BiHADA. SEED includes three emotional categories, namely positive, negative, and neutral emotions. The dataset includes 45 sets of EEG data collected from 15 participants at three different sessions, using a 62-channel ESI NeuroScan system. For each collection experiment, 15 video clips corresponding to positive, negative, and neutral emotions were used to induce emotional responses. Each segment has a duration of approximately 4 min and is manually edited to maximize the emotional induction effect of the video material. Among them, positive, negative, and neutral emotions correspond to five segments each. There is a 5-s hint before the segment, followed by a self-assessment after approximately 4 min, and a 15-s rest time before playing the next segment. The paradigm of collection experiment is shown in Figure 4.

SEED-IV and SEED-V belong to the same series as SEED. SEED-IV has four emotional categories, namely happiness, sadness, fear, and neutral emotions. SEED-IV contains 45 sets of EEG data collected from 15 participants at three different time periods. In the collection experiment, each emotion category corresponds to 6 video materials, so in each experiment, participants need to watch a total of 24 video clips, which means 24 trials. SEED-V includes EEG data from 16 participants at three different time periods, with a total of five emotional categories: happy, fearful, disgust, neutral, and sad emotions.

### 3.2. Data Preprocessing

The EEG data was down-sampled to 200 Hz. To reduce noise and eliminate artifacts, a bandpass filter was applied, resulting in signals extracted across five frequency bands: δ (1–3 Hz), θ (4–7 Hz), α (8–13 Hz), β (14–30 Hz), and γ (31–50 Hz) [23]. After preprocessing, each channel’s EEG data is segmented using a 1-s time window without overlap, and based on this, we extracted the DE features of the EEG signals on five bands. Differential entropy (DE) features are commonly used in fields such as signal processing, image processing, and bioinformatics [24]. The differential entropy feature takes into account the dynamic changes of the data sequence, so it can better capture the dynamic nature of the data compared to traditional static entropy features. Due to the fact that EEG signals have higher low-frequency energy than high-frequency energy, differential entropy has the ability to distinguish the balance of EEG signal patterns between low-frequency and high-frequency energy.

The EEG data is segmented based on the midline of the brain where the reference electrode in the 10–20 system is located, and the differential entropy features are extracted. The differential entropy features are horizontally stacked in series according to the electrode arrangement of each hemisphere.

Finally, the data format of the left and right hemispheres in the SEED dataset is 3 (sessions) × 15 (subjects) × 3394 (samples) × 27 (channels) × 5 (frequency bands). The number of samples in different periods in the SEED-IV dataset is slightly different, and the data format of the left and right hemispheres is approximately 3 (sessions) × 15 (subjects) × 822 (samples) × 27 (channels) × 5 (frequency bands). SEED-V includes EEG data of 16 subjects in three sessions, and sample numbers for each subject in three periods are different, 681 (samples), 541 (samples) and 601 (samples), respectively.

To avoid information leakage during preprocessing, normalization was performed independently for each subject or each session before constructing the source and target domains. Specifically, the normalization statistics were computed within the corresponding subject/session data rather than jointly across the source and target domains. No target domain labels were used during normalization, and source domain and target domain samples were not mixed to compute shared normalization statistics. Therefore, the preprocessing procedure did not introduce label information from the target domain into model training.

### 3.3. Experimental Protocol

The effectiveness of the BiHADA model was evaluated using the Leave-One-Subject-Out (LOSO) method. In the cross-subject experiment, for each trial, one of the subjects’ data was selected as the target domain, with the data from the other subjects serving as source domain. The process was iterated for each subject, resulting in a total of SES(sessions) × SUB(subjects) experiments. The accuracy was recorded at the end of each experiment, and the average accuracy was calculated across all trials.

In the cross-session experiment, for each trial, two sessions’ data from the selected subject were used as the source domain, with the remaining data serving as the target domain. The process was iterated for each session, resulting in a total of SUB × SES experiments. Similarly, the accuracy was recorded at the end of each experiment, and the accuracy was calculated across all trials.

### 3.4. Experimental Settings

The left- and right-hemisphere branches are both implemented using a multilayer perceptron architecture, consisting of a feature extractor, an emotion classifier, and a class-informed discriminator. For each hemisphere-specific branch, the model input is a 135-dimensional DE feature vector, where 135 is obtained by concatenating the features from 27 hemisphere-specific electrodes across five frequency bands. The feature extractor first maps the input feature from 135 dimensions to 128 dimensions, and then further projects it into a 64-dimensional latent feature space. Batch normalization and LeakyReLU activation are applied after each linear layer to improve training stability and enhance nonlinear representation capability.

The resulting 64-dimensional feature representation is further processed by batch normalization and LeakyReLU activation, and is then fed into the emotion classifier and the class-informed discriminator, respectively. The emotion classifier consists of two fully connected layers, which map the 64-dimensional feature representation to a 32-dimensional hidden representation and finally output the prediction over C emotion categories. The class-informed discriminator adopts a similar two-layer fully connected structure, mapping the 64-dimensional feature representation to a 32-dimensional hidden representation and producing a C + 1-dimensional discrimination output. The first C output nodes correspond to the source domain emotion categories, while the additional node represents the target domain class. LogSoftmax is adopted in the output layers of both the classifier and the discriminator.

During model training, the Adam optimizer is used with an initial learning rate of 0.01. The batch size is set to 32, and the number of training epochs is set to 200. The trade-off parameter λ  in the loss function is used to balance the source domain emotion classification loss and the class-informed adversarial discrimination loss. In this study, λ  is treated as a fixed hyperparameter rather than being dynamically scheduled during training.

To provide a more rigorous statistical evaluation of the experimental results, statistical analyses were performed on the trial-wise accuracies obtained from the same evaluation trials. For each ablation study, six comparisons were conducted, corresponding to two evaluation protocols, namely cross-subject and cross-session experiments, on three datasets. To control the family-wise error rate caused by multiple comparisons, the Holm–Bonferroni correction was applied to the obtained *p*-values. Therefore, the *p*-values reported in this study refer to Holm–Bonferroni-adjusted *p*-values. A corrected *p*-value lower than 0.05 was considered statistically significant.

## 4. Result

### 4.1. Cross-Subject and Cross-Session

Table 1 presents the average accuracies of the cross-subject experiments conducted by the BiHADA model across three sessions, as well as the average accuracies of the cross-session experiments.

BiHADA-L and BiHADA-R represent the ADA-CiD branch networks trained separately using left hemisphere data and right hemisphere data, respectively. BiHADA uses the perplexity obtained from both branch networks to compute the classifier’s weight and achieve the final category prediction through the label decider.

The BiHADA model proposed in this section demonstrates excellent performance across three datasets in the SEED series. In the cross-subject experiments, BiHADA achieved average accuracies of 86.82%, 65.78%, and 71.43%, respectively. In the cross-session experiments, the average accuracies were 92.71%, 68.24%, and 74.35%, respectively, all outperforming the BiHADA-L and BiHADA-R models that use independent hemisphere data.

On SEED and SEED-IV, BiHADA-R achieved average accuracies of 83.37% and 63.91% in cross-subject experiments, showing improvements of 4.38% and 1.50% compared to BiHADA-L, with a more noticeable difference in accuracy on SEED. In cross-session experiments, BiHADA-R achieved average accuracies of 91.56% and 67.76%, showing improvements of 2.33% and 0.40% compared to BiHADA-L, with smaller differences in accuracy in cross-session experiments. Across these three datasets, BiHADA-R consistently outperformed BiHADA-L in average accuracy, indicating that due to the asymmetry of brain, emotional responses differ between hemispheres. Moreover, right hemisphere data demonstrated a significant advantage in both experiments.

**Figure 5:**
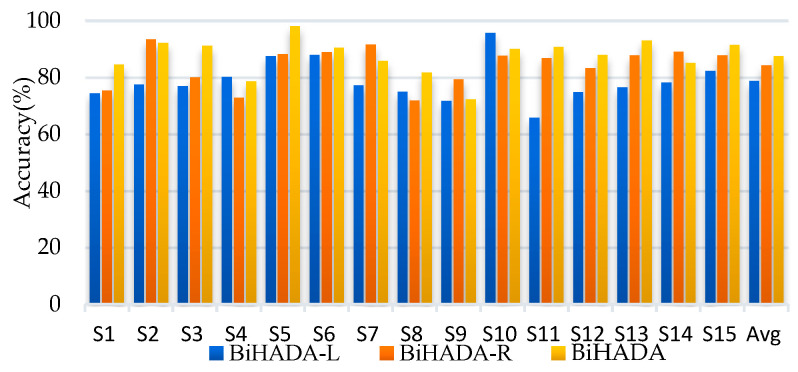
Classification accuracy of SEED cross-subject experiment on session 1.

The BiHADA network, which integrates data from both hemispheres for comprehensive decision-making, demonstrates a significant improvement in accuracy across all experiments compared to the branch network BiHADA-R that models using only right hemisphere data. Although right hemisphere data outperforms left hemisphere data in emotional classification, the method proposed in this section, BiHADA, achieves higher accuracy by leveraging branch networks based on both hemispheres data. This indicates that the branch networks modeled on the left and right hemispheres data are complementary in the process of emotion recognition using EEG. Figure 4 and Figure 5 illustrate the accuracy of each subject in the cross-subject experiment on session 1 and the cross-session experiment targeting session 1, respectively, for the three models.

**Figure 6:**
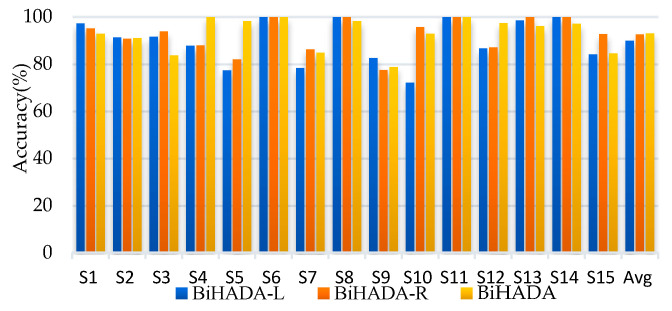
Classification accuracy of SEED cross-session experiment on session 1.

It can be seen from Figure 5 and Figure 6 that there are notable differences between BiHADA-L and BiHADA-R in accuracy across different subjects. The average cross-subject accuracy of BiHADA-L is 5.50% lower than that of BiHADA-R, while the average cross-session accuracy differs by 2.70%. The right hemisphere data demonstrates higher accuracy in both emotion classification tasks and achieves a lower standard deviation compared to the left hemisphere data. This suggests that the emotional lateralization mechanism affects the accuracy of emotion classification.

Compared to BiHADA-L, BiHADA achieves an accuracy improvement of 8.78% in the cross-subject task and 3.20% in the cross-session task. It achieves an accuracy improvement of 3.28% and 0.46% when compared to BiHADA-R. BiHADA demonstrates a more pronounced enhancement in classification performance compared to BiHADA-L, which only utilizes data from the left brain hemisphere.

Overall, the proposed method achieves a higher average accuracy in the cross-subject task on session 1 compared to the average accuracy across all three sessions. In the cross-session task, the average accuracy when session 1 is the target domain also surpasses the overall average accuracy. This suggests that the data from session 1 is more conducive to the proposed model learning discriminative representations. While ensuring the indistinguishability of features between domains, the features within each domain exhibit stronger specificity.

### 4.2. Comparison with Other Methods

The comparison of classification accuracy between BiHADA and other emotion recognition models on the SEED dataset is shown in Table 2. To ensure a fair comparison, all methods adopted the same preprocessing procedure, feature extraction method, evaluation protocol, and training–testing partition as those used in this study. The compared models are described as follows.

Transfer Component Analysis (TCA) learns transfer components by maximizing the mean difference across domains to reduce domain discrepancies [25].

Domain Confusion for Deep Learning (DCORA) is a deep domain adaptation method that incorporates the CORAL loss function into neural networks to minimize differences in cross-domain feature covariance [26].

Domain-Adversarial Neural Network (DANN) is a classic unsupervised deep domain adaptation method that employs adversarial training to learn feature representations with domain invariance [10].

Dynamical Graph Convolutional Neural Networks (DGCNNs) model multi-channel EEG features using graphs convolutional neural networks to perform emotion classification [27].

TMLP is used to extract emotion-related EEG features, and SRDANN serves as the domain adaptation module, focusing on source domain samples with stronger transferability through sample weighting [28].

BiDANN includes three domain discriminators, with two local domain discriminators learning discriminative emotional features for the corresponding hemispheres. Another global domain discriminator is used to reduce overall domain discrepancies [17].

Transferable Attention Neural Network (TANN) adaptively highlights transferable EEG data and samples using local and global attention mechanisms to learn discriminative emotional information [29].

Cross-subject EEG-based emotion recognition through dynamic optimization of random forest with sparrow search algorithm (SSA-RF) is a classical machine-learning method for cross-subject EEG emotion recognition that uses the sparrow search algorithm to optimize the key hyperparameters of a Random Forest, and then selects effective feature combinations for emotion classification [30].

Joint Distributed Instances Represent Transfer (JD-IRT) is a transfer-learning method for EEG emotion recognition that combines Joint Distribution Deep Adaptation (JDDA) with Instance-Representation Transfer (I-RT) to simultaneously reduce marginal and conditional distribution discrepancies and to select more suitable source domain samples for transfer [31].

Bipartite Graph Adversarial Network (BP-Graph) is a subject-independent EEG emotion recognition method that incorporates bipartite graphs into a DANN-style adversarial framework to further reduce inter-subject variability and learn more domain-invariant emotional features [32].

### 4.3. Frequency Band Analysis

To investigate the effect of different frequency bands on the classification accuracy of the model, the model BiHADA was employed to conduct emotion EEG recognition for different frequency band data on session 1 of the SEED dataset. The cross-subject experimental results of each frequency band are all presented in Table 3.

For specific subjects, different frequency bands showed different emotional recognition effects. Among them, the δ frequency band had the lowest average accuracy rate across subjects in the cross-subject task, which was 63.86%, while the γ frequency band had the highest average accuracy rate, which was 76.72%. In the comparison of low-frequency bands (δ, θ, α), the θ frequency band had the highest accuracy rate, reaching 70.71%, indicating that in the low-frequency signals, the θ frequency band is more conducive to the model extracting emotion EEG features with cross-domain invariance. Overall, the high-frequency bands (β, γ) had an 8.50% and 12.86% increase in accuracy rate compared to the δ frequency band respectively, demonstrating a significant advantage in the emotion EEG recognition task, indicating that the feature separability of the high-frequency bands is relatively strong.

Table 4 shows the emotional recognition accuracy rates of 15 participants in the cross-period experiments, with the data from period 1 serving as the target domain, as well as the standard deviations of the accuracy rates for the same participants across different frequency bands. For some participants in period 1, the accuracy rates showed relatively small differences, such as participants S06, S09, etc., with the lowest standard deviation being 5.08%. There were also some participants who demonstrated significant differences in the accuracy rates of different frequency bands in the emotional recognition task, such as participants S03 and S04, whose standard deviations in different frequency bands were 14.72% and 14.90%, respectively. Combining the results of Table 3 and Table 4, it indicates that the adaptability of different frequency bands to the emotional EEG recognition task varies. Overall, when considering only a single frequency band, the high-frequency bands (β, γ) are more suitable for cross-subject and cross-period tasks. In contrast, the EEG features of the high-frequency bands have a stronger correlation with emotional processing.

The BiHADA model is evaluated on the SEED dataset across three sessions under both cross-subject and cross-session settings, and the average accuracy and standard deviation are reported. Differential entropy features extracted from different frequency bands and their concatenated combinations are used as inputs, and the results are shown in Table 5. When using full-band features, the model achieves an average accuracy of 86.82% with a standard deviation of 8.09. When the concatenation of δ, θ, α, and β bands is used as input to BiHADA, the average accuracy is 82.70%, which is 4.12% lower than that of the full-band setting. However, this configuration uses fewer frequency bands and achieves a comparable standard deviation of 8.41, indicating similar stability. In the three-band concatenation experiments, the highest accuracy is achieved by the θ, β, and γ combination, reaching 81.74%, which is 5.08% lower than the full-band performance. In the two-band concatenation experiments, the best-performing combination is β and γ, with an average accuracy of 79.23%, which is 7.59% lower than that of the full-band setting.

The full-band results for the cross-session experiments are presented in Table 6, where BiHADA achieves an average accuracy of 92.71%. Compared with the cross-subject setting, the overall average accuracy in the cross-session experiments is consistently higher. This indicates that the impact of the non-stationarity of EEG signals on the model is smaller than that caused by inter-subject variability. Consequently, the emotional patterns in EEG data under cross-session settings are easier for the model to learn and capture.

### 4.4. Weight Analysis

**Figure 7:**
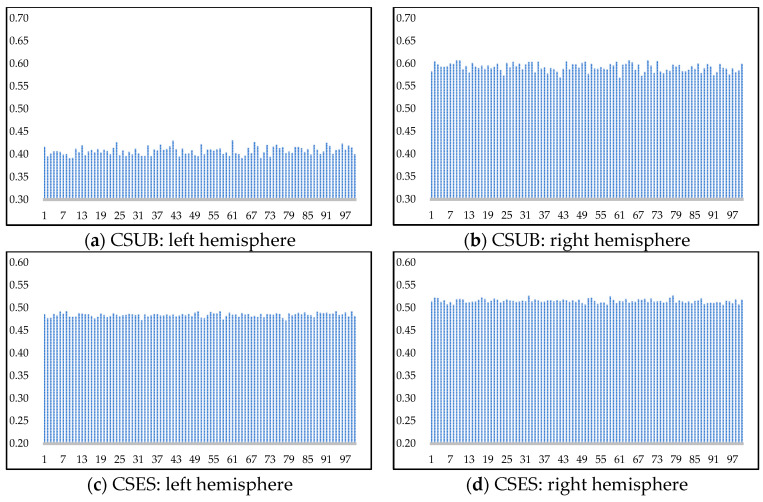
Weights of the left and right hemispheres in cross-subject (CSUB) and cross-session (CSES) experiments on SEED dataset.

To investigate the impact of the branch networks of different hemispheres on the category prediction by the label decider, this section randomly selected one participant from the SEED dataset for cross-domain experiments, analyzing the classifier weights for their left and right hemispheres. The results are shown in Figure 7, where the horizontal axis denotes sample batches, each containing 32 sample data, and the vertical axis represents the weights.

Figure 7a,b display the weights of the different hemispheres across different batches in the cross-subject experiments. The average weight for the right hemisphere in category decision-making is 0.592, indicating a smaller difference in feature distribution between both domains of the right hemisphere in the cross-subject experiments. This higher weight, computed via perplexity, suggests a greater impact of the right hemisphere’s branch network predictions on label decision-making.

Figure 7c,d show the weights of the different hemispheres across different batches in the cross-session experiments. The average weight difference between both hemispheres is 0.031, indicating a similar degree of feature distribution difference between domains of the left and right hemispheres in the cross-session experiments. Data from three sessions are used for source and target domain partitioning. Due to the data originating from the same subject, the initial distribution shift is small. After domain adaptation processing, both left and right hemisphere data achieve good distribution alignment, resulting in similar weights for both hemisphere classifiers in label decision-making. In contrast, in the cross-subject experiments, due to individual differences in EEG signals, there is a larger initial distribution shift between domains, and the degree of marginal distribution alignment of left and right hemisphere data also shows a significant difference. In the proposed BiHADA model in this section, right hemisphere data exhibit better domain adaptation effects, resulting in higher weights in label decision-making.

### 4.5. Parameter Sensitivity Analysis

**Figure 8:**
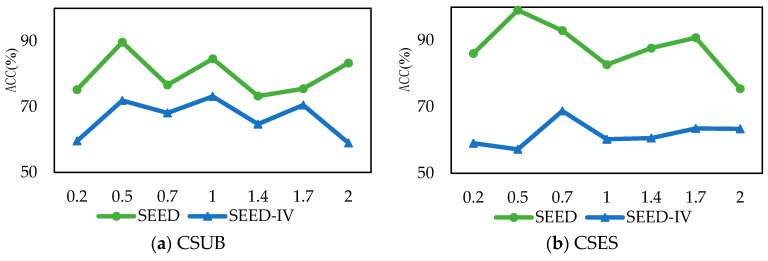
The influence of parameters on classification accuracy.

In order to study the influence of the hyperparameter settings in BiHADA on the classification performance of the model, this subsection conducts a parameter sensitivity test on the weight parameter λ of $\;L\left(\theta_{f},\;\theta_{y},\theta_{d}\right)$ in Equation (17), and performs experimental verification on SEED and SEED-IV. The results are shown in Figure 8.

The cross-subject emotion EEG classification experiment selected the data of subject S01 as the target domain. The results are shown in Figure 8a. In the experiment of the SEED dataset, when the λ value was 0.5, BiHADA achieved the highest classification accuracy. In the experiment of the SEED-IV dataset, when the λ value was 1, BiHADA achieved the highest classification accuracy. In the experiments where λ was greater than 1, the classification accuracy decreased. The cross-period emotion EEG classification experiment selected the data of period 2 of subject S01 as the target domain. The experimental results are shown in Figure 8b. In the experiments of the SEED and SEED-IV datasets, when λ was 0.5 and 0.7 respectively, the best classification accuracy was achieved. Based on the above experimental results, it indicates that when the λ value is between 0.5 and 1, the model BiHADA proposed in this chapter can achieve a relatively high emotion classification accuracy.

### 4.6. Perturbation Analysis

**Figure 9:**
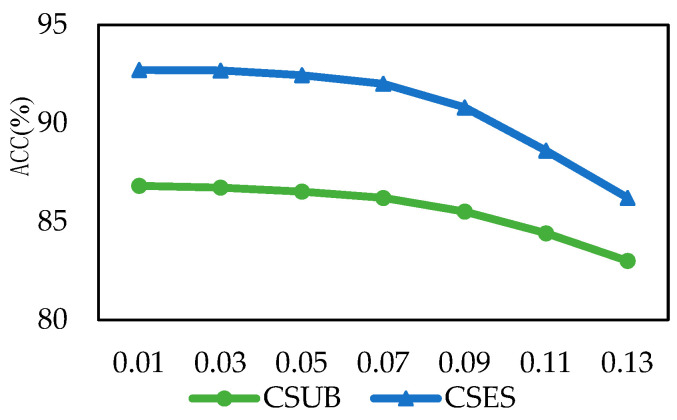
The influence of Gaussian noise on classification accuracy.

To validate the robustness of the proposed BiHADA model under noise interference, Gaussian noise with different intensities was injected into the input features, and the model performance was evaluated under both cross-subject and cross-session settings. The results are shown in Figure 9.

Overall, as the noise intensity increases, the classification accuracy of BiHADA in both tasks gradually decreases. However, the degradation remains relatively smooth, without abrupt performance collapse, indicating that the proposed model maintains good stability and robustness under noise perturbation. In particular, within the low-to-moderate noise range, the performance remains relatively stable, suggesting that the proposed method has a strong resistance to a certain degree of data contamination. In addition, BiHADA does not exhibit severe performance breakdown in either task after noise injection, which further demonstrates that its bi-hemispheric modeling and weighted decision mechanism remain effective in noisy scenarios. On the one hand, the left and right hemisphere branches extract features separately, which helps preserve complementary emotional representations from different hemispheres; when one hemisphere branch is more strongly affected by noise, the other branch may still provide relatively stable discriminative information. On the other hand, the weighting strategy based on branch adaptation quality can reduce the negative influence of the branch that is more severely disturbed by noise during the final decision stage, thereby improving the stability of prediction. Therefore, even as the noise level gradually increases, BiHADA is still able to maintain a relatively smooth degradation trend, demonstrating good robustness against noise perturbation.

### 4.7. Training Data Size Analysis

**Figure 10:**
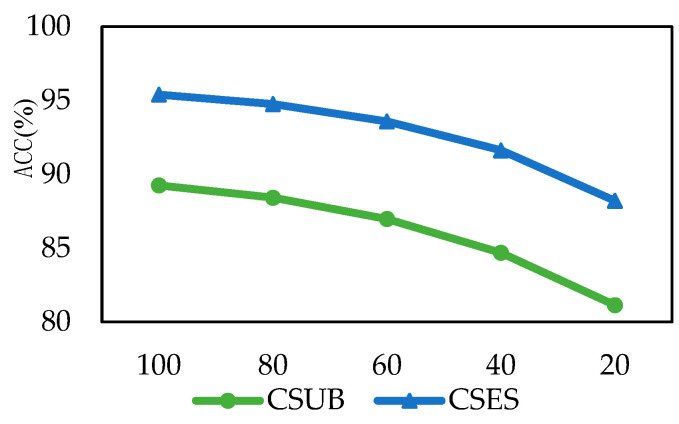
The influence of training set size on classification accuracy.

To further evaluate the stability and generalization ability of the proposed BiHADA model under limited training data, reduced-training-data experiments were conducted in this section. Under both the cross-subject and cross-session settings, the test-set partition strategy was kept the same as that in the main experiments, and only the size of the source domain training set was adjusted. Specifically, in the cross-subject experiments, one subject was fixed as the target domain test set, while the remaining subjects were used as the source domain training set. In the cross-session experiments, one session was fixed as the target domain test set, and the remaining sessions were used as the source domain training set. On this basis, different proportions of the source domain training data were randomly retained, with training data ratios set to 20%, 40%, 60%, 80%, and 100%. Here, 100% denotes the use of all source domain training data, while the other ratios indicate that the training set was constructed by random subsampling of the source domain data while keeping the target domain test set unchanged. By comparing the classification accuracies under different training data ratios, the robustness of the model under limited training samples was evaluated. The results of the reduced-training-data experiments are shown in Figure 10.

In the cross-subject experiments, when the training data ratio was gradually reduced from 100% to 80%, 60%, 40%, and 20%, the accuracy decreased from 89.25% to 88.41%, 86.97%, 84.68%, and 81.14%, respectively. In the cross-session experiments, the accuracy decreased from 95.40% to 94.76%, 93.58%, 91.62%, and 88.21%, respectively. These results show that, in both the cross-subject and cross-session scenarios, BiHADA was able to maintain relatively high recognition accuracy under moderate reductions in training data, indicating that the model has a certain adaptability to mild-to-moderate decreases in training data. Even when only 20% of the training data were used, BiHADA still achieved accuracies of 81.14% and 88.21% in the cross-subject and cross-session tasks, respectively, demonstrating that the proposed method retains good recognition capability under low-resource conditions. This result is closely related to the bi-hemispheric modeling and weighted decision mechanism of BiHADA. On the one hand, the left and right hemisphere branches are modeled separately, which helps extract complementary emotional discriminative information from different hemispheres and thus preserves relatively rich feature representation capability even when the number of training samples is reduced. On the other hand, the weighting strategy based on branch adaptation quality can dynamically integrate the outputs of the two hemisphere branches during the final classification stage, thereby alleviating the instability caused by insufficient training data in a single branch and improving the overall robustness of the model under limited training data conditions.

### 4.8. Ablation on Class-Informed Discriminator

To validate the effect of the class-informed discriminator on model performance, this section compares the class-informed discriminator in BiHADA with a binary domain discriminator. The variant employing the binary domain discriminator is denoted as BiHADA-BD, and comparative experiments are conducted on the SEED, SEED-IV, and SEED-V datasets. The results are presented in Table 7. Overall, BiHADA outperforms BiHADA-BD in most experimental settings. To further determine whether these performance gains are statistically significant, paired statistical tests were conducted on the trial-wise accuracies obtained from the same evaluation trials.

On the SEED dataset, the accuracy of BiHADA increases from 86.27% to 86.82% in the cross-subject experiment and from 92.27% to 92.71% in the cross-session experiment, while the standard deviation in the cross-subject experiment decreases from 9.85 to 8.09, indicating that the class-informed discriminator helps improve both recognition accuracy and model stability. Moreover, the *p*-values for both the cross-subject and cross-session experiments on SEED are lower than 0.05, demonstrating that the improvements are statistically significant. On the SEED-V dataset, the advantage of BiHADA is more pronounced: compared with BiHADA-BD, its cross-subject and cross-session accuracies are improved by 0.58% and 1.96%, respectively, while the standard deviations decrease from 16.03 and 14.47 to 13.63 and 12.44. The *p*-values for both experiments on SEED-V are lower than 0.03, indicating that these improvements are also statistically significant. These results suggest that, in experiments with more categories and more complex cross-domain discrepancies, the class-informed discriminator can more effectively enhance feature alignment quality.

In contrast, on the SEED-IV dataset, the accuracy of BiHADA is slightly lower than that of BiHADA-BD, indicating that the class-informed mechanism does not consistently bring performance gains under all data distributions and experimental settings. This result also suggests that the introduction of class-structure constraints may be affected by dataset characteristics, emotion-category complexity, and the degree of cross-domain distribution shift. One possible reason is that SEED-IV may involve more complex distribution overlap among different emotion categories or stronger cross-domain variations, making the adversarial constraint based on source domain class structure less stable for some target domains and potentially increasing optimization difficulty. Therefore, the class-informed discriminator is not interpreted as being universally superior to the binary domain discriminator. Instead, it is considered to have the potential to improve class-level alignment in most experimental settings. Together with the results on SEED and SEED-V, these findings suggest that the class-informed discriminator can help preserve category-discriminative information and alleviate cross-category misalignment under certain conditions, but its effectiveness remains dependent on the specific data distribution and the complexity of the cross-domain task.

### 4.9. Ablation on Perplexity

To verify the effectiveness of the perplexity-weighted strategy, this section conducts an ablation study on whether to introduce perplexity weighting, and the comparison results are shown in Table 8. Across the three datasets, under both cross-subject and cross-session settings, the BiHADA model with perplexity weighting denoted as BiHADA-NP, consistently outperforms the unweighted variant in overall performance. To further assess whether these performance gains are statistically significant, paired statistical tests were conducted on the trial-wise accuracies obtained from the same evaluation trials.

On the SEED dataset, compared with the unweighted variant, BiHADA improves the accuracy by 0.24% and 0.21% in the cross-subject and cross-session tasks, respectively, while the standard deviation is also reduced, indicating improved model stability. The corresponding statistical analysis shows that the *p*-values for both the cross-subject and cross-session tasks on the SEED dataset are below 0.042, demonstrating that these improvements are statistically significant. On the SEED-IV dataset, BiHADA also achieves higher average accuracy in both tasks, together with lower standard deviations, suggesting that perplexity weighting helps mitigate distribution discrepancies under both cross-subject and cross-session settings. The paired statistical tests further confirm that the *p*-values for both tasks are below 0.046. On the SEED-V dataset, although the improvement in average accuracy is relatively modest, the model still exhibits better stability, and the statistical results indicate that the *p*-values for both tasks are below 0.038, showing statistical significance.

**Figure 11:**
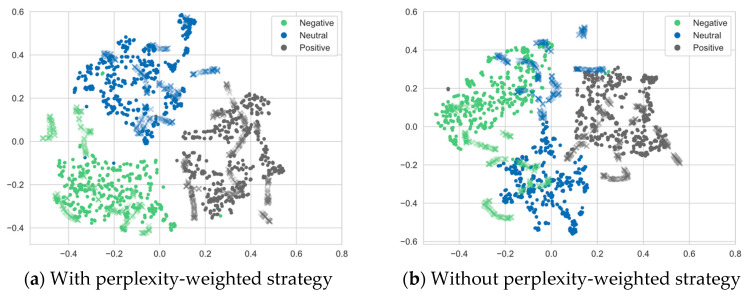
Visualization of target domain feature distributions with and without the perplexity-weighted strategy. Circles represent the source domain and crosses represent the target domain.

As shown in Figure 11, a two-dimensional visualization of the target domain features is provided to further analyze the effect of the perplexity-weighted strategy on feature discriminability. Figure 11a shows the feature distribution with the perplexity-weighted strategy, while Figure 11b shows the feature distribution without this strategy. It can be observed that, after introducing the perplexity-weighted strategy, the features of different emotion categories become more compact and the inter-class boundaries are clearer. In particular, the overlap among negative, neutral, and positive samples is reduced. In contrast, without perplexity weighting, more obvious feature mixing and class overlap can be observed, and some samples are distributed more scatteredly, indicating weaker category-discriminative structures. These results suggest that the perplexity-weighted strategy can dynamically adjust the contribution of different hemisphere branches according to their adaptation quality on the target domain, allowing better-aligned and more reliable branches to contribute more to the final prediction. Consequently, this strategy enhances the class separability of target domain features and improves the stability and generalization capability of the model.

Overall, the perplexity-weighted strategy adjusts the contribution of different branches according to their estimated adaptation quality on the target domain. In this way, branches with relatively better alignment are assigned larger weights in the final decision. The experimental results suggest that this strategy may contribute to the performance improvement of BiHADA, although its effect varies across datasets and evaluation protocols.

### 4.10. Ablation on Feature Extractor

To validate the effectiveness of hemisphere-specific feature extractors, this section replaces the independent feature extractors in the left and right branches of BiHADA with a shared feature extractor, and denotes the resulting variant as BiHADA-SC. Comparative experiments were then conducted on the SEED, SEED-IV, and SEED-V datasets. The results show that BiHADA overall outperforms BiHADA-SC. To further determine whether these performance gains are statistically significant, paired statistical tests were conducted on the trial-wise accuracies obtained from the same evaluation trials. The comparison results are shown in Table 9.

On the SEED dataset, the accuracy of BiHADA increases from 86.32% to 86.82% in the cross-subject task and from 92.40% to 92.71% in the cross-session task, while the corresponding standard deviations decrease from 10.00 and 8.89 to 8.09 and 8.34, respectively. These results indicate that the use of independent feature extractors not only improves recognition accuracy but also enhances model stability. The corresponding statistical analysis further shows that the *p*-values for both the cross-subject and cross-session tasks on the SEED dataset are below 0.031, indicating that these improvements are statistically significant. On the SEED-V dataset, compared with BiHADA-SC, BiHADA improves the accuracy by 0.35% in the cross-subject task and by 0.19% in the cross-session task, while the standard deviations decrease from 13.95 and 12.91 to 13.63 and 12.44, respectively. This suggests that hemisphere-specific feature modeling can still provide stable performance gains in scenarios with more emotion categories and more complex emotional patterns. The statistical results further show that the *p*-values for both tasks on the SEED-V dataset are below 0.048, indicating that these improvements are also statistically significant. On the SEED-IV dataset, BiHADA achieves only slightly higher accuracy than BiHADA-SC in the cross-subject task, and the performance gain in the cross-session task is also relatively limited, indicating that the relative advantage of shared versus independent feature extractors may depend on the specific data distribution and experimental setting.

The performance difference between the two variants may be related to the different assumptions made by the feature extractors. A shared feature extractor assumes that the left and right hemispheres follow a similar feature mapping pattern. Although this design can reduce the number of model parameters and may strengthen the modeling of common characteristics, it may not fully capture the functional differences between the two hemispheres in emotional processing. In contrast, the independent feature extractors adopted in BiHADA allow the left and right hemispheres to learn hemisphere-specific representations separately. This design may help preserve emotional lateralization characteristics and provide more suitable features for subsequent class-level domain alignment and classification. However, it should also be noted that the performance gains of BiHADA over BiHADA-SC are relatively modest in some settings. Therefore, these results suggest that independent feature extractors can provide a certain benefit within the bi-hemispheric domain adaptation framework.

### 4.11. Feature Visualization Analysis

**Figure 12:**
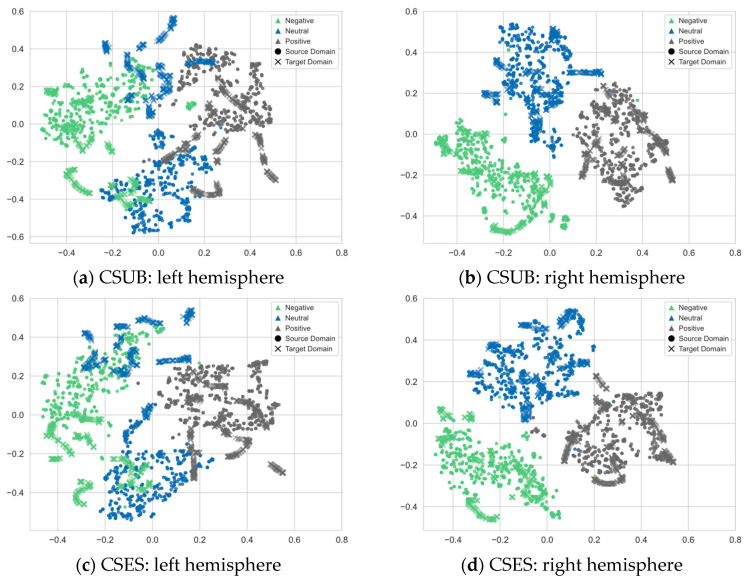
Domain adaptation feature visualization of the left and right hemisphere in cross-subject (CSUB) and cross-session (CSES) experiments on SEED dataset.

To further analyze the differences in domain adaptation between left and right hemispheres data, this section employs the t-SNE algorithm on the SEED dataset to visualize the domain-invariant features of both brain hemispheres after adversarial domain adaptation. The visualized feature distributions are presented in Figure 12, where different colors represent three emotions: negative, neutral, and positive. The domains are differentiated using two different shapes, where circles denote the source domain and crosses the denote target domain.

Figure 12a,b depict the feature visualization outcomes from the cross-subject experiment, where one subject is randomly selected as target domain, while the remaining 14 subjects are considered as the source domain. Figure 12a depicts the visualization results of left hemisphere features. From the perspective of domain adaptation, it can be observed that each emotion features in both domains are aligned to some extent at the class level. However, there is noticeable overlap between features of negative and neutral emotions, while positive emotions in the left hemisphere exhibit better separability compared to the other two. Figure 12b presents the visualization results of right hemisphere features in the cross-subject experiment. The right hemisphere features exhibit clearer classification boundaries, with only a small number of misalignments between features of negative and neutral emotions. Overall, the right hemisphere demonstrates a higher level of domain adaptation, with smaller marginal distribution differences between both domain data and more significant specificity of different emotional categories, resulting in stronger separability.

Figure 12c,d depict the feature visualization results of the cross-session experiment, where the data from session 1 of a randomly selected subject is considered as target domain, while data from the other two sessions are considered as source domain. Figure 12c,d depict the visualization results of both hemispheres features of this subject. Compared to the cross-subject experiment, the cross-session experiment exhibits smaller differences in feature distribution and clearer classification boundaries. In the cross-session experiment, the classification boundaries of right hemisphere features are clearer than those of left hemisphere features. The alignment of features from both the left and right hemispheres is higher in the cross-session experiment compared to the cross-subject experiment.

### 4.12. Confusion Matrix Analysis

**Figure 13:**
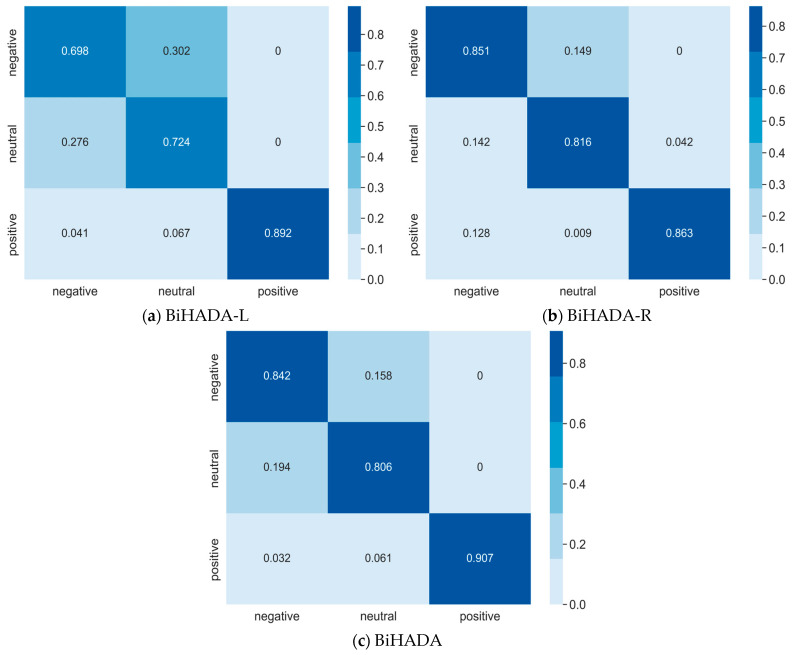
Confusion matrix of BiHADA and its branch networks.

Due to the emotional lateralization mechanism, both hemispheres exhibit differences in response to different emotional stimuli. To investigate the perception abilities of different hemisphere regions towards different emotional patterns and validate the effectiveness of BiHADA, this section conducts a cross-subject experiment on SEED dataset. One subject is randomly selected as target domain, with the other subjects serving as source domain. The emotion classification abilities of the left and right hemisphere branch networks, as well as the emotion classification ability of the BiHADA model integrating information from both hemispheres, are compared using confusion matrices, as shown in Figure 13.

Figure 13a presents the classification results of the network BiHADA-L modeled using data from the left hemisphere. The left hemisphere tends to confuse negative and neutral emotions, while exhibiting a higher classification accuracy for positive emotions.

Figure 13b displays the emotion classification results of the network BiHADA-R modeled using data from the right hemisphere. The right hemisphere demonstrates a significantly higher classification accuracy for negative emotions compared to the left hemisphere. Moreover, it achieves an accuracy of 81.6% in the classification of neutral emotions, showing an advantage over the left hemisphere. However, the accuracy for positive emotions is lower compared to the left hemisphere.

Figure 13c illustrates the label prediction results of BiHADA after weighting the classifier outputs from the left- and right-hemisphere branch networks. BiHADA achieves accuracies of 84.2% and 80.6% in the classification experiments of negative and neutral emotions, respectively, showing a significant improvement in classification performance compared to the results obtained from the left hemisphere data. In the classification task of positive emotions, BiHADA achieves an accuracy of 90.7%, surpassing the accuracies of the branch networks from both hemispheres.

In summary, the left and right hemispheres exhibit distinct emotional perceptions, with the left hemisphere showing a stronger correlation with positive emotions and the right hemisphere being more closely related to negative emotions, consistent with findings from neuroscience research. Additionally, in the experiments conducted in this section, the right hemisphere demonstrates stronger recognition abilities for neutral emotional patterns.

## 5. Conclusions

In order to exploit multimodal information in data alignment and capture the asymmetry of brain hemispheres’ responses to emotions, this paper proposes a Bi-Hemispheric Adversarial Domain Adaptation Neural Network. First, a class-informed discriminator is developed by opening the label structure information of the source domain data. Perplexity is then used to quantify the marginal distribution differences between features from the adversarial branch networks corresponding to the left and right hemispheres. This metric is used to assign the weights to the classifiers, mitigating negative knowledge transfer from misaligned sample features during the final classification. Comparative experiments show that the use of the class-informed discriminator and distinct hemisphere branch networks significantly improves classification performance. Future research should investigate domain adaptation using multiple source domains to develop an emotion recognition model with enhanced adaptability and generalization, considering the prevalence of diverse source domains in practical scenarios.

Although the proposed BiHADA method achieved promising performance on the SEED, SEED-IV, and SEED-V datasets, this study still has several limitations. First, the scale of the evaluated datasets is relatively limited, as these EEG emotion datasets contain a small number of subjects and recording sessions. This may restrict the generalizability of the experimental conclusions to larger and more diverse populations. Second, the proposed method was evaluated only on publicly available benchmark datasets from the SEED series. External validation on independent EEG datasets collected with different devices, experimental paradigms, emotional stimuli, or subject populations is still needed to further verify the robustness and general applicability of the model. Third, although the main experimental settings and model architecture have been described, reproducibility may still be affected by implementation details such as random initialization, software and hardware environments, and dataset preprocessing differences. In future work, we will further evaluate the proposed method on larger-scale and multi-center EEG datasets, provide more complete implementation details and source code, and explore more standardized evaluation protocols to improve the reproducibility and practical applicability of the model.

## Figures and Tables

**Table 1:** Classification accuracy on SEED, SEED-IV, and SEED-V, CSUB represents cross-subject, and CSES represents cross-session.

Model	SEED		SEED-IV		SEED-V	
	CSUB	CSES	CSUB	CSES	CSUB	CSES
BiHADA-L	78.99 (10.32)	89.23 (10.53)	62.41 (10.78)	67.36 (13.13)	67.78 (16.78)	68.27 (18.25)
BiHADA-R	83.37 (9.46)	91.56 (7.57)	63.91 (11.24)	67.76 (12.04)	68.89 (17.09)	69.91 (17.34)
BiHADA	86.82 (8.09)	92.71 (8.34)	65.78 (10.31)	68.24 (11.90)	71.43 (13.63)	74.35 (12.44)

**Table 2:** Comparison with other advanced methods on SEED.

Model	TCA [25]	DCORAL [26]	DANN [10]	DGCNN [27]
Acc (Std)	63.64 (14.88)	68.25 (7.85)	77.62 (9.12)	79.95 (9.02)
Model	TMLP+ SRDANN [28]	BiDANN [17]	TANN [29]	BiHADA
Acc (Std)	81.04 (6.28)	83.28 (9.60)	84.41 (8.75)	86.82 (8.09)
Model	SSA-RF [30]	JD-IRT [31]	BP-Graph [32]	
Acc (Std)	78.44 (12.06)	83.03 (7.25)	85.9 (6.71)	

**Table 3:** Cross-subject classification accuracy across different frequency bands on the SEED dataset.

Subject	Frequency Bands					
	δ	θ	α	β	γ	SD
S01	69.72	83.38	78.45	63.01	85.64	9.51
S02	83.05	76.18	71.04	76.68	82.66	5.02
S03	97.24	68.43	69.02	84.64	95.82	13.94
S04	65.10	83.28	56.73	91.07	94.81	16.58
S05	60.28	98.37	88.15	92.14	89.00	14.71
S06	98.74	99.55	88.46	97.84	99.97	4.79
S07	75.74	65.74	72.50	79.48	85.22	7.32
S08	65.37	81.83	68.04	75.43	88.70	9.64
S09	65.14	70.89	65.17	68.06	68.23	2.42
S10	76.27	65.12	63.46	85.04	91.25	12.14
S11	64.73	84.64	89.34	74.00	79.47	9.57
S12	71.58	89.19	72.62	98.65	91.83	12.08
S13	84.24	94.42	98.47	94.80	98.41	5.82
S14	84.06	99.08	90.5	99.96	98.04	6.85
S15	83.41	76.81	68.12	68.72	88.88	9.07
AVG	76.31	82.46	76.47	83.30	89.20	9.30

**Table 4:** Cross-session classification accuracy across different frequency bands on the SEED dataset.

Subject	Frequency Bands					
	δ	θ	α	β	γ	SD
S01	69.41	88.05	79.39	62.85	82.63	10.19
S02	85.85	75.62	71.44	78.24	81.40	5.45
S03	98.26	64.99	69.79	86.70	94.10	14.72
S04	65.17	84.10	62.06	90.61	94.81	14.90
S05	65.42	99.16	86.41	99.58	92.04	14.02
S06	97.67	99.83	87.47	98.97	99.97	5.29
S07	76.40	72.31	69.4	80.41	84.20	5.97
S08	65.48	75.40	59.54	74.55	88.73	11.10
S09	67.42	72.63	75.11	69.04	80.21	5.08
S10	78.93	64.78	78.26	82.06	89.67	9.02
S11	67.04	85.11	88.52	75.40	75.86	8.54
S12	71.55	82.47	75.95	99.75	94.04	11.92
S13	85.04	95.00	98.40	94.38	99.55	5.71
S14	84.25	99.12	92.65	98.05	98.38	6.27
S15	87.30	76.59	65.71	73.24	97.60	12.49
AVG	77.68	82.34	77.34	84.25	90.21	9.38

**Table 5:** Classification accuracy of cross-subject experiments with different frequency-band combinations on the SEED dataset.

δ	θ	α	β	γ	δ + θ	δ + α
64.65 (11.30)	69.72 (12.41)	65.82 (13.14)	73.90 (10.47)	74.49 (8.37)	75.16 (8.22)	68.47 (11.05)
δ + β	δ + γ	θ + α	θ + β	θ + γ	α + β	α + γ
75.12 (9.13)	74.47 (8.70)	75.66 (8.46)	77.02 (8.49)	79.26 (9.50)	73.41 (8.53)	76.54 (10.23)
β + γ	δ + θ + α	δ + θ + β	δ + θ + γ	δ + α + β	δ + α + γ	δ + β + γ
79.23 (9.08)	78.64 (8.32)	76.17 (11.54)	82.13 (8.93)	79.08 (8.25)	80.49 (9.50)	79.04 (8.29)
θ + α + β	θ + α + γ	θ + β + γ	α + β + γ	δ + θ + α + β	θ + α + β + γ	δ + θ + α + β + γ
80.92 (8.53)	80.42 (8.27)	81.74 (9.37)	81.15 (9.66)	82.70 (8.41)	81.03 (9.78)	86.82 (8.09)

**Table 6:** Classification accuracy of cross-session experiments with different frequency-band combinations on the SEED dataset.

δ	θ	α	β	γ	δ + θ	δ + α
77.17 (13.26)	83.40 (11.04)	76.83 (13.57)	84.29 (10.88)	87.45 (10.46)	88.93 (8.68)	83.26 (10.44)
δ + β	δ + γ	θ + α	θ + β	θ + γ	α + β	α + γ
87.06 (9.11)	85.34 (8.05)	87.25 (8.84)	88.11 (8.12)	86.95 (10.17)	86.76 (9.02)	87.41 (8.60)
β + γ	δ + θ + α	δ + θ + β	δ + θ + γ	δ + α + β	δ + α + γ	δ + β + γ
88.90 (9.14)	89.03 (7.60)	87.58 (8.49)	90.26 (7.48)	90.45 (6.82)	90.12 (7.51)	88.04 (8.55)
θ + α + β	θ + α + γ	θ + β + γ	α + β + γ	δ + θ + α + β	θ + α + β + γ	δ + θ + α + β + γ
88.25 (8.17)	88.46 (8.29)	88.60 (8.75)	89.72 (8.78)	90.68 (7.42)	90.87 (6.65)	92.71 (8.34)

**Table 7:** Classification accuracy between BiHADA-BD and BiHADA on the SEED dataset.

Model	SEED		SEED-IV		SEED-V	
	CSUB	CSES	CSUB	CSES	CSUB	CSES
BiHADA-BD	86.27 (9.85)	92.27 (8.27)	67.95 (10.84)	69.73 (11.66)	70.85 (16.03)	72.39 (14.47)
BiHADA	86.82 (8.09)	92.71 (8.34)	65.78 (10.31)	68.24 (11.90)	71.43 (13.63)	74.35 (12.44)

**Table 8:** Classification accuracy between BiHADA-NP and BiHADA on the SEED dataset.

Model	SEED		SEED-IV		SEED-V	
	CSUB	CSES	CSUB	CSES	CSUB	CSES
BiHADA-NP	86.58 (9.96)	92.50 (8.72)	65.72 (11.06)	68.22 (11.84)	71.15 (14.24)	74.29 (12.77)
BiHADA	86.82 (8.09)	92.71 (8.34)	65.78 (10.31)	68.24 (11.90)	71.43 (13.63)	74.35 (12.44)

**Table 9:** Classification accuracy between BiHADA-SC and BiHADA on the SEED dataset.

Model	SEED		SEED-IV		SEED-V	
	CSUB	CSES	CSUB	CSES	CSUB	CSES
BiHADA-SC	86.32 (10.00)	92.40 (8.89)	65.48 (11.27)	68.13 (12.33)	71.08 (13.95)	74.16 (12.91)
BiHADA	86.82 (8.09)	92.71 (8.34)	65.78 (10.31)	68.24 (11.90)	71.43 (13.63)	74.35 (12.44)

## Data Availability

The SEED and SEED-IV dataset are all publicly available. The SEED dataset is available at https://bcmi.sjtu.edu.cn/home/seed/seed.html, accessed on 29 June 2024. The SEED-IV dataset is available at https://bcmi.sjtu.edu.cn/home/seed/seed-iv.html, accessed on 29 June 2024. The SEED-V dataset is available at https://bcmi.sjtu.edu.cn/home/seed/seed-v.html, accessed on 29 June 2024.
